# The frequency of pulmonary hypertension in juvenile scleroderma

**DOI:** 10.1186/1546-0096-12-S1-P307

**Published:** 2014-09-17

**Authors:** Amra Adrovic, Kenan Barut, Aida Koka, Refet Gojak, Tuncalp Demir, Funda Oztunc, Ozgur Kasapcopur

**Affiliations:** 1 Pediatric Rheumatology, Istanbul University, Cerrahpasa Medical Faculty, Istanbul, Turkey; 2 Pediatric Cardiology, Istanbul University, Cerrahpasa Medical Faculty, Istanbul, Turkey; 3 Cardiology, Sarajevo University, Sarajevo, Bosnia and Herzegovina; 4 Chest Diseases, Istanbul University, Cerrahpasa Medical Faculty, Istanbul, Turkey

## Introduction

Juvenile scleroderma (JS), represents a rarely seen group of connective tissue disease with multiple organ involvement. Although quite rare in childhood, cardio-vascular and pulmonary involvements are the most important mortality and morbidity factors. Pulmonary arterial hypertension (PAH), the most important sequelae of pulmonary involvement, could be determined by echocardiographic examinations. Early cardio-vascular and pulmonary involvement determination is extremely important in reducing mortality of patients.

## Objectives

The aim of the study was to use non-invasive methods (echocardiography, pulmonary function tests) to examine cardio-pulmonary involvement of the disease in patients. Treatment of patients with positive findings in the early stage of the disease possibly reduces the morbidity and mortality.

## Methods

Totally of 35 patients with scleroderma, followed up at Cerrahpasa Medical Faculty, Pediatric Rheumatology Department with diagnosis of juvenile scleroderma were included in the study. Doppler echocardiography was performed at Cerrahpasa Medical Faculty, Pediatric Cardiology Department and pulmonary function tests were performed at Laboratory for pulmonary function tests at Cerrahpasa Medical Faculty. FVC and DLCO were measured in order to investigate pulmonary fibrosis. The assessment of PAP and risk factors for PAH was made by measurement of maximum tricuspid insufficiency (TI), end diastolic pulmonary insufficiency (PI), AT/ET, RAP and contraction of vena cava inferior during inspiration.

## Results

The values of TI, PI, AT/ET and PAP were found to be normal and statistically signıficant different from the pathological values. The results of FVC and DLCO were found to be statistically significant above normal values. In other words, no patient was found to have cardio-pulmonary involvement.

## Conclusion

Although quite rare in juvenile scleroderma, cardio-vascular and pulmonary involvement is the most important factor in the prognosis of the disease. Early diagnosis, regular follow up and appropriate treatment are important in reducing the cardio-vascular and pulmonary complications of the disease.

## Disclosure of interest

None declared.

